# Molecular Classification of Endometrial Carcinoma: Protocol for a Cohort Study

**DOI:** 10.2196/34461

**Published:** 2022-08-04

**Authors:** Inês Moreira, Carla Bartosch, Manuel Teixeira, Marta Ferreira

**Affiliations:** 1 Oncology Department Instituto Português de Oncologia do Porto Porto Portugal; 2 Pathology Department Instituto Português de Oncologia do Porto Porto Portugal; 3 Genetics Department Instituto Português de Oncologia do Porto Porto Portugal

**Keywords:** endometrial carcinoma, molecular classification, prognosis, POLE, mismatch repair, p53

## Abstract

**Background:**

Endometrial carcinoma (EC) is the most common gynecologic malignancy in developed countries and the fourth most frequent in women worldwide. The cornerstone of treatment for EC is surgery. Clinicopathological features are currently used to help determine the individual risk of recurrence and the need for adjuvant treatment after surgery. Nonetheless, there is significant interobserver variability in assigning histologic subtype when using a morphological classification, revealing the need for a more unified approach. The Cancer Genome Atlas (TCGA) project identified 4 distinct prognostic EC subtypes based on genomic abnormalities. Surrogate assays including 3 immunohistochemical markers (p53, MSH6, and PMS2) and 1 molecular test (mutation analysis of the exonuclease domain of DNA polymerase epsilon; *POLE*) allowed the development and validation of a simplified molecular classifier that correlates with the TCGA classification, has prognostic value, and can easily be used in clinical practice. This molecular classification categorizes EC in 4 subtypes: *POLE* mutated, mismatch repair–deficient, p53 abnormal, and no specific molecular profile. Applying this classification in clinical practice will help tailor adjuvant treatment decisions.

**Objective:**

The aim of this study is to retrospectively apply this novel molecular classification to a cohort of patients with EC treated in a comprehensive cancer center, to assess its applicability in clinical practice, to evaluate clinical outcomes by molecular subtypes, and to assess its prognostic value.

**Methods:**

In this retrospective cohort study, patients with primary EC diagnosed during and after 2013 and treated or followed at our institution, after definite surgery, will be included. Demographic and clinicopathological data will be obtained from electronic health records and from pathology reports. Laboratory methods will include immunohistochemical study of p53 and mismatch repair proteins, as well as *POLE* mutational analysis by genetic sequencing. The primary end point is recurrence-free survival and secondary end points are disease-specific survival and overall survival. A descriptive analysis of variables will be carried out. Survival analysis will be performed using the Kaplan-Meier method and the groups will be compared using the log-rank test.

**Results:**

This protocol was reviewed and approved by the Instituto Português de Oncologia do Porto, Portugal, ethics committee in October 2021; patient selection from our cancer registry began the same month. A total of 160 patients will be included. This work will present real-life results that will allow a better understanding of the Portuguese EC population and the distribution of the molecular subgroups throughout. We will use these results to understand the prognostic value of this classification in our population and its role in adjuvant therapy decisions. This study is anticipated to conclude in December 2022.

**Conclusions:**

This study will provide important information regarding these women’s outcomes according to this new molecular classification and will support its use when discussing a patient’s need for adjuvant treatment.

**International Registered Report Identifier (IRRID):**

PRR1-10.2196/34461

## Introduction

### Background

Endometrial carcinoma (EC) is the most common gynecologic malignancy in developed countries and the fourth most frequent in women worldwide [1]. Incidence of EC has been increasing in the past several years, mainly due to an increase in obesity rates, which is one of the most important risk factors for this disease [2]. Other conditions associated with metabolic syndrome, including diabetes mellitus and polycystic ovary syndrome, and conditions involving excess estrogen exposure such as estrogen-producing tumors or tamoxifen use (which has antiestrogenic effects in the breast and proestrogenic effects in the uterus) are other known risk factors. Protective factors against EC include multiparity and oral contraceptive use [2]. Lynch syndrome, an inherited disorder caused by germline mutations in DNA mismatch repair genes, accounts for approximately 3% of all endometrial cancers [2]. Women with mutations in *MLH1*, *MSH2*, *MSH6,* or *PMS2* have up to a 40%-60% lifetime risk of developing both endometrial and colorectal cancers, as well as a 9%-12% lifetime risk of developing ovarian cancer [3].

The cornerstone of treatment for EC is surgery, consisting of a total hysterectomy and bilateral salpingo-oophorectomy [4]. Most patients with EC present with early-stage, low-grade disease that has a low risk of recurrence and can be managed by surgery alone. Clinicopathological features including age, International Federation of Gynaecology and Obstetrics (FIGO) stage [4], depth of myometrial invasion, tumor differentiation grade, histopathologic tumor type (endometrioid, serous, clear cell, undifferentiated carcinoma, carcinosarcoma, mixed), and lymphovascular space invasion help determine the individual risk of recurrence and the need for adjuvant treatment after surgery [5]. A risk group classification to guide adjuvant therapy decisions was proposed by the European Society of Medical Oncology (ESMO) 2013 clinical practice guidelines and was updated at the ESMO 2016 consensus conference [6,7]. Nonetheless, there is significant interobserver variability in assigning histologic subtype when using this morphological classification, revealing the need for a more unified approach. Moreover, there are many unanswered questions regarding EC’s optimal management, including which, if any, adjuvant therapies to administer.

The Cancer Genome Atlas (TCGA) endometrial collaborative project identified 4 distinct prognostic EC subtypes based on genomic abnormalities, raising the possibility of more precise tailoring of adjuvant therapy [8]. These 4 subgroups include DNA polymerase epsilon (*POLE*) ultra-mutated (with a very high mutation burden in the exonuclease domain of *POLE* that leads to its inactivation and failure in proofreading during DNA replication), microsatellite instability (MSI) hypermutated, copy-number low, and copy-number high. This molecular classification correlates with patient prognosis and may help to improve the identification of early-stage patients who may benefit from adjuvant therapy. However, these genomic methodologies, including genome sequencing, are expensive and can be complex, when obtaining DNA from frozen tissue.

In an effort to bring this molecular classification to routine clinical practice, a reproducible and cost-effective approach that correlated to the TCGA classification was proposed [9]. A simplified molecular classifier was developed, which identifies four molecular subtypes that are analogous to the four genomic subgroups described in TCGA:

1. Pathogenic mutations in the exonuclease domain of *POLE* (*POLE* mutated; *POLE*mut) corresponding to the POLE ultra-mutated phenotype
2. Mismatch repair–deficient (MMRd), with altered immunohistochemical expression of mismatch repair proteins (MMR), corresponding to the MSI hypermutated group
3. No specific molecular profile (NSMP), with preserved p53 and MMR immunohistochemical expression, corresponding to the copy-number low group, having a low mutational burden
4. p53 abnormal (p53abn), with aberrant p53 immunohistochemical expression, including complete loss and/or overexpression of p53, corresponding to the copy-number high group, with a high mutational burden

This classification is based on surrogate simple molecular assays used in clinical practice that could replicate the TCGA classification: MMR immunohistochemistry (IHC) assay (MLH1, MSH2, MSH6 and PMS2) to identify MMRd, genetic sequencing for *POLE* exonuclease domain mutations, and an IHC assay for p53 (wild type vs mutation-type expression; p53wt and p53abn, respectively). These tools can be used in standard formalin-fixed paraffin-embedded (FFPE) material.

Different working groups achieved replication of TCGA survival curves with statistical significance using this molecular classification. These results were further confirmed and validated in other patient cohorts, establishing this simple molecular classifier as a prognostic marker for progression-free and disease-specific survival [10,11]. Tumors with *POLE*mut (~10% of ECs) were mainly of the endometrioid type and had very favorable prognosis, and p53abn tumors (~11%) were associated with aggressive tumor characteristics and consisted mostly of high-grade serous ECs, with poor outcomes. MMRd tumors (28%) and NSMP tumors (~51%) were also mostly endometrioid ECs and had an intermediate prognosis.

The majority of EC can be classified into 1 of the 4 molecular subgroups. However, in a small subset of patients (3%-5%), molecular analysis will show more than one classifying alteration, also referred to as “multiple-classifier” EC [12,13]. The prognosis of these “multiple-classifier” ECs is still uncertain but available survival data demonstrated that *POLE*mut-p53abn EC shows clinical outcomes comparable to *POLE-*mutated EC without abnormal p53 expression and that MMRd–p53abn shows clinical outcomes comparable to MMRd without abnormal p53 expression [13].

The prognostic value of the molecular classification shown in the previous studies was explored in a retrospective combined analysis of the PORTEC-1 and PORTEC-2 cohorts [14]. These authors concluded that molecular analysis was feasible in >96% of the patients and also reported an unfavorable prognosis for the p53abn group, an intermediate prognosis for the MSI and NSMP groups, and a favorable prognosis in the *POLE*-mutated group. This classification was further investigated in the high-risk patient cohort of the PORTEC-3 trial, and the impact of adjuvant treatment for each molecular subgroup was also evaluated [15]. In this cohort, molecular analysis was successful in 97% of patients; the authors concluded that the molecular classification has a strong prognostic value in high-risk EC (again showing an excellent outcome for *POLE*-mutated patients and a worse outcome for p53abn patients, with the MSI and NSMP groups having an intermediate outcome), and that patients with p53abn EC should be considered for adjuvant chemoradiotherapy, whereas for those with *POLE*-mutated ECs, de-escalation of adjuvant treatment should be considered. These two retrospective analyses further support the incorporation of this molecular classification in the risk stratification of patients with EC, as well as in future trials, with the aim of reducing both overtreatment and undertreatment. Moreover, applying this classification in clinical practice will led to personalized treatment approaches based on molecular risk groups and may help tailor immunotherapy in patients with EC [16].

The more recent European Society of Gynaecological Society/European SocieTy for Radiotherapy and Oncology/European Society of Pathology (ESGO/ESTRO/ESP) guidelines of 2021 for the management of patients with EC recommend using the molecular classification in all ECs, especially high-grade and/or high-risk tumors, and it should be integrated with traditional pathologic features to define prognostic risk groups [17].

To determine the optimal adjuvant treatment within each molecular subtype, this molecular-based classification should be incorporated in future clinical trials to improve outcomes for women with EC; for example, molecular-integrated classification is currently being investigated in the PORTEC-4a trial and in the RAINBO umbrella program (Refining Adjuvant treatment IN endometrial cancer Based On molecular profile) [18,19].

### Objectives

The aim of this study is to analyze the distribution of the molecular subtypes in patients with EC treated at our cancer center (Instituto Português de Oncologia do Porto, Portugal; IPO-Porto) and their respective correlation with histopathological and patient characteristics, to assess its applicability in clinical practice, to compare molecular subtypes and ESMO risk groups, and to evaluate clinical outcomes by molecular subtype and assess the prognostic value of this molecular classification. We also aim to evaluate the behavior and clinical evolution of patients with tumors categorized as multiple-classifier, a subject where information is still scarce, in order to better understand their prognosis.

## Methods

### Study Design

This is a retrospective cohort study of patients with primary EC, diagnosed during and after 2013 and treated or followed at IPO-Porto, after definite surgery.

This study will be carried out by the Medical Oncology department of IPO-Porto, in collaboration with the Pathology and Genetics departments. The Medical Oncology department will be responsible for the selection of patients, gathering and analysis of data, and elaboration of the final report. Pathology will oversee the molecular analysis via IHC in tumor specimens. The investigation of *POLE*mut will be carried out by Genetics. All departments participated in the study design.

### Participants and Cohort Identification

This study will include women aged ≥18 years with written informed consent, histological diagnosis of primary EC, definitive surgical staging performed, surgery specimen available at IPO-Porto (for molecular analysis), and availability of clinicopathological and outcome data.

Patients with the following characteristics will be excluded: concurrent cancer being treated at the same time as EC; any neoadjuvant treatment; and metastatic (stage FIGO IVB) and advanced disease (stage FIGO III – IVA with residual tumor).

Patients will be selected from the IPO-Porto cancer registry and from the diagnostic database of the Pathology department. We will start by selecting all women with an EC diagnosis during and after 2013, and evaluate each patient according to the inclusion and exclusion criteria. Afterward, this database will be sent to the Pathology department to check for tumor sample availability.

With a power of 80% and a maximum probability of type 1 error of 5%, the target sample size is estimated to be 160 patients. With this target sample size, we estimate that we will have sufficient power to find statistically significant differences in the study’s primary and secondary end points.

### Clinical and Laboratory Data Collection

Demographic and clinicopathological data will be obtained from electronic health records and from pathology reports. As for laboratory methods for p53 and MMR protein IHC study and *POLE* mutational analysis, data will be collected as described below.

#### p53 and MMR IHC

A representative FFPE tissue block will be selected for p53 and MMR protein IHC study. IHC assays will be performed on FFPE tissue sections using a Leica Bond-III automated staining instrument according to the manufacturer’s instructions. The following antibodies, clones, titers, and vendors will be used: p53 (Clone D07, 1:200, Dako), MLH1 (Clone ES05, 1:150, Leica), MSH2 (Clone 25D12, 1:150, Leica), MSH6 (Clone PU29, 1:200, Leica), and PMS2 (Clone MOR4G, 1:50, Leica). Immunostained slides will be evaluated by a pathologist using the following classification: p53 wild type expression (ie, multifocal expression); p53 mutation type/aberrant expression, which includes complete absence of expression, cytoplasmic expression, and overexpression; MMR-proficient tumors (ie, those with intact MMR protein expression); and MMR-deficient tumors (ie, those showing patterns of MMR expression that include complete loss, subclonal loss, or weak immunoexpression).

#### POLE Mutation Analysis

Tissue sections from selected FFPE tissue blocks will be used for tumor macrodissection and DNA extraction. Genomic DNA will be submitted to polymerase chain reaction amplification followed by Sanger sequencing, using primers for the exonuclease domain of the *POLE* (exons 9-14) gene. *POLE* variants will be described according to LRG_789t1 (NM_006231.4) and the Human Genome Variation Society guidelines [20].

The following information will be collected for posterior data analysis: patient-related variables (age, body mass index, Eastern Cooperative Oncology Group performance status at diagnosis, date of diagnosis); tumor-related variables (histological subtype, tumor grade, FIGO 2009 stage, lymphovascular space invasion, myometrial invasion, nodal status, ESMO clinical risk groups, pelvic or aortic lymphadenectomy, adjuvant treatment performed, type of adjuvant treatment, *POLE* exonuclear domain mutations, p53 IHC status, MMR IHC status, molecular subtype [*POLE*mut, MMRd, NSMP, p53abn, multiple-classifier]); outcome-related variables (recurrence of disease, location of metastasis, death, cause of death, status at last follow-up visit, date at last follow-up visit).

### Study End Points

The primary end point of this study is recurrence-free survival, defined as the time from the date of surgery until recurrence of disease documented by the attending physician. Secondary end points are disease-specific survival, defined as the time from the date of surgery until death due to EC, and overall survival, defined as the time from the date of surgery until death from any cause.

### Statistical Analysis

Descriptive analysis of variables will be carried out. Continuous variables will be presented using quantitative measures (median, median, quartiles, minimum and maximum values, and standard deviation). Categorical variables will be presented as frequencies and percentages. The Kolmogorov-Smirnov test will be used to verify the normality of the data.

Comparisons between groups will be performed, using Mann-Whitney and Kruskal-Wallis tests for continuous variables. Chi-square or Fisher exact tests will be used to evaluate the association between categorical variables, when appropriate. Survival analysis to assess the main outcomes over time will be performed using the Kaplan-Meier method and the groups will be compared using the log-rank test. Univariable and multivariable analysis with Cox regression models will be used to control the survival analysis according to relevant factors.

A *P* value <.05 will be considered significant. Statistical analysis will be conducted using the SPSS software (version 27; IBM Corp).

### Data Availability and Collection

In October 2021, after ethics committee approval (see below), patient selection from the IPO-Porto cancer registry began. This information will then be sent to the Pathology department to verify tumor material availability. Afterward, the selection of patients’ tumor samples will be completed and data collection and laboratory analysis will begin.

### Ethics Approval

This protocol was reviewed and approved by the IPO-Porto ethics committee in October 2021 (CES IPO: 233/021). Informed consent will be collected. This study will be conducted according to the principles of the Helsinki Declaration [21]. All collected information will be processed anonymously and used solely for the purposes of this protocol.

## Results

Laboratory analysis is scheduled to start after all inclusion data have been collected, and is expected to be concluded by October 2022. Final data analysis will proceed, with an aim to publish a peer-reviewed paper divulging results by the end of 2022.

This protocol will be submitted for grant applications to several entities that support clinical and translational research to obtain funding.

## Discussion

### Principal Findings

Molecular classification and its prognostic value were validated based on retrospective studies. Its prognostic value was further explored in a retrospective analysis of cohorts of patients from previous randomized trials that included patients with EC. These studies support the incorporation of this molecular classification in the risk stratification of patients with EC, as well as in future trials, with the aim of reducing both overtreatment and undertreatment. Based on these results, the ESGO/ESTRO/ESP guidelines published in January 2021 [17] recommended the use of this molecular classification in all ECs, especially high-grade and/or high-risk tumors, and that it should be integrated with traditional anatomopathological features to define prognostic risk groups. Prospective trials incorporating this molecular classification are currently in progress [18,19] and its application in clinical practice is only just starting.

Our study will present real-life results that will allow a better understanding of the Portuguese EC population and the distribution of the molecular subgroups in this population. We will use these results to understand the prognostic value of this classification in our population and its role in adjuvant therapy decisions. We anticipate that our findings will correlate to previously published studies [9-11]. However, these studies were conducted in North America and northern Europe, so differences in the molecular profile of this southern European population might be expected.

### Strengths

Information obtained from this study will help tailor adjuvant therapy in patients with EC according to molecular subgroups and introduce this molecular classification into our center’s clinical practice. We now know from the studies that validated this molecular classification that, despite traditional clinicopathological features, patients with *POLE* mutations have an extremely good prognosis and therefore may not need adjuvant treatment (even if they have high-risk histological features that would make them candidates for adjuvant treatment according to current practice). On the other hand, patients with p53 abnormal IHC status are associated with worse prognosis, and so adjuvant therapy should be mandatory. Patients in the MMRd and NSMP subgroups have an intermediate prognosis and adjuvant therapy decisions should be individualized. Applying this molecular classification to the decision of whether to proceed with adjuvant treatment will help reduce undertreatment and overtreatment and therefore reduce patient morbidity related to treatment toxicities and health care–related costs.

Moreover, we hope to characterize clinicopathological and molecular features of multiple-classifier EC, which is an area of the literature where information is lacking, and understand the evolution and prognosis of these multiple-classifier tumors. We believe this study will provide clinically relevant data for the management of EC. This study will also enable the identification of potential Lynch syndrome patients, who warrant specific surveillance.

### Limitations

Besides being a retrospective analysis, this study has some limitations. There is a possibility of having to exclude various patients if there are no tumor samples available, which might decrease follow-up time. Moreover, due to budget constraints, we will not be able to use next-generation sequencing, as previous groups have used, to determine the presence of *POLE* mutations, and we will be using Sanger sequencing instead. Although this might pose a challenge to correlating our findings with work already published, we will be able to evaluate how this method behaves for this particular purpose and perhaps establish Sanger sequencing as a more economical alternative to next-generation sequencing, despite it being more time-consuming.

### Conclusions

Molecular classification provides prognostic information that impacts the management of patients with EC. However, its use in clinical practice is only just beginning and results from prospective trials are eagerly awaited. This study will provide important information regarding these women’s outcomes according to this new molecular classification and will support its use when discussing a patient’s need for adjuvant treatment. Ultimately, the use of this classification will reduce treatment-related morbidity and health care–related costs.

## Abbreviations

EC: endometrial carcinoma
ESGO: European Society of Gynaecological Society
ESMO: European Society of Medical Oncology
ESP: European Society of Pathology
ESTRO: European SocieTy for Radiotherapy and Oncology
FFPE: formalin-fixed paraffin-embedded
FIGO: International Federation of Gynaecology and Obstetrics
IHC: immunohistochemistry
IPO-Porto: Instituto Português de Oncologia do Porto, Portugal
MMR: mismatch repair proteins
MMRd: mismatch repair–deficient
MSI: microsatellite instability
NSMP: no specific molecular profile
p53abn: p53 abnormal
POLE: DNA polymerase epsilon
POLEmut: POLE mutated
RAINBO: Refining Adjuvant treatment IN endometrial cancer Based On molecular profile
TCGA: The Cancer Genome Atlas
